# Manifestation of Hydrogen Bonding and Exciton Delocalization on the Absorption and Two-Dimensional Electronic Spectra of Chlorosomes

**DOI:** 10.1021/acs.jpcb.2c07143

**Published:** 2023-01-25

**Authors:** Vesna Erić, Xinmeng Li, Lolita Dsouza, Sean K. Frehan, Annemarie Huijser, Alfred R. Holzwarth, Francesco Buda, G. J. Agur Sevink, Huub J. M. de Groot, Thomas L. C. Jansen

**Affiliations:** † University of Groningen, Zernike Institute for Advanced Materials, 9747 AG Groningen, The Netherlands; ‡ Department of Chemistry and Hylleraas Centre for Quantum Molecular Sciences, University of Oslo, Sem Sælands vei 26, 0315 Oslo, Norway; § Leiden Institute of Chemistry, Leiden University, Einsteinweg 55, 2300 RA Leiden, The Netherlands; ∥ MESA+ Institute for Nanotechnology, University of Twente, Drienerlolaan 5, 7522 NB Enschede, The Netherlands; ⊥ Department of Biophysical Chemistry, Max Planck Institute for Chemical Energy Conversion, Stiftstraße 34-36, 45470 Mülheim, Germany

## Abstract

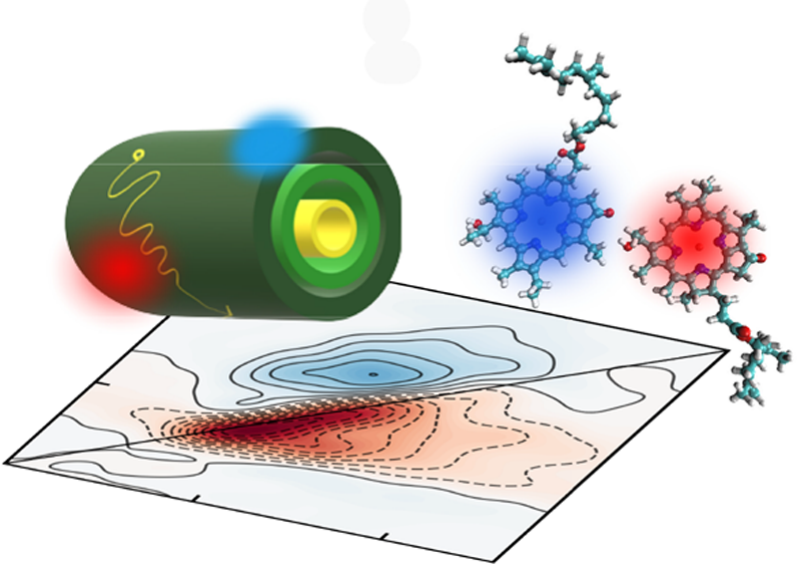

Chlorosomes are supramolecular aggregates that contain thousands of bacteriochlorophyll molecules. They perform the most efficient ultrafast excitation energy transfer of all natural light-harvesting complexes. Their broad absorption band optimizes light capture. In this study, we identify the microscopic sources of the disorder causing the spectral width and reveal how it affects the excited state properties and the optical response of the system. We combine molecular dynamics, quantum chemical calculations, and response function calculations to achieve this goal. The predicted linear and two-dimensional electronic spectra are found to compare well with experimental data reproducing all key spectral features. Our analysis of the microscopic model reveals the interplay of static and dynamic disorder from the molecular perspective. We find that hydrogen bonding motifs are essential for a correct description of the spectral line shape. Furthermore, we find that exciton delocalization over tens to hundreds of molecules is consistent with the two-dimensional electronic spectra.

## Introduction

**Figure 1:**
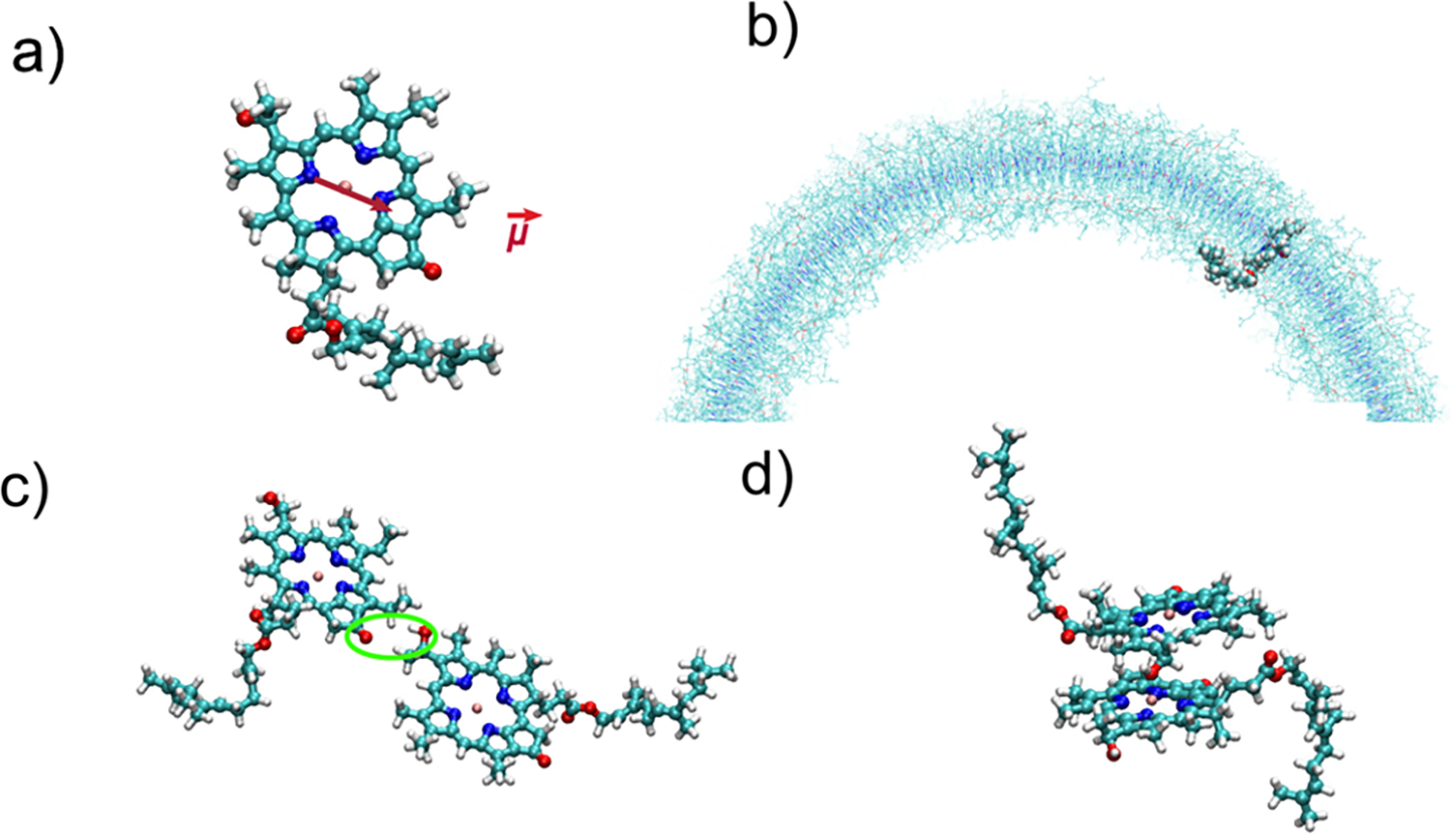
a) Structure of the BChl *c* molecule, main building block of chlorosomes, with the transition dipole moment of the Q_*y*_ displayed as a red vector. b) View from top on the atomistic structure of one of the tubular aggregates which shows how BChl *c* molecules are embedded in the environment of other chromophores. The supramolecular structure is maintained through c) hydrogen bonding and d) π–π parallel stacking of alternating *syn*-*anti* BChl *c* molecules stabilized by Mg–O coordination.

*Chlorobaculum tepidum* is a green sulfur bacterium that thrives in extremely low-light conditions owing to the evolutionary optimization and adaptation of its photosynthetic architecture.^1,2^ Their light-harvesting apparatus consists of a chlorosome antennae, an organelle that absorbs the light, the baseplate, and the Fenna–Matthews–Olson complex funneling the light energy to the reaction center.^3−5^ Chlorosomes are known to perform the most efficient ultrafast excitation energy transfer among all natural light-harvesting complexes.^6,7^ Interestingly, their structure is unique compared to all other known photosynthetic components since it consists almost exclusively of closely packed chromophores and there is no need for protein scaffolding to ensure stability of the complex.^3^ Chlorosomes are in the order of 100–150 nm long with diameters ranging from 20 to 50 nm.^5,8^ Each organelle contains around 10^5^ bacteriochlorophyll (BChl) *c/d/e* molecules. The distinct structure of the chlorosomal aggregates and their efficiency as a light-harvesting antennae suggest an intricate relationship. Even though these properties inspired the design of artificial light-harvesting systems,^9,10^ the effect of the structural inhomogeneities and dynamics on the functional properties of the natural chlorosomes is still not entirely understood. Complete characterization of their structure, which is significantly more heterogeneous compared to other light-harvesting complexes, requires combining the information from different methods. Experimental evidence from cryo-electron microscopy (cryo-EM),^11^ solid-state nuclear magnetic resonance (NMR) spectroscopy,^12^ X-ray,^8^ and optical spectroscopies^13^ lead to assignment of molecular packing. Bacteriochlorophyll molecules form alternating *syn-anti* parallel stacks^12^ that are stabilized through hydrogen bonding, π–π stacking interactions, and Mg–O coordination, as represented in Figure 1. The presence of interstack hydrogen bonding interactions leads to formation of helical secondary structures within the self-assemblies.

Interplay of these intermolecular interactions leads to formation of supramolecular structures for which two different types of geometries are proposed: assemblies of concentric cylinders^11^ and lamellae.^8^ For the tubular aggregates, different angles for rolling up the sheets have been reported.^12−15^ In this manuscript, we will present a microscopic model that allows us to predict the linear absorption and two-dimensional electronic spectra and reveal the origin of the structural heterogeneity and dynamics responsible for the spectral linebroadening. We will add additional mesoscopic scale disorder originating from the variation in tube dimensions (primarily radii) and orientations^7,16^ in a phenomenological way.

Although every chlorosome is unique as the details of the structural organization will differ depending both on the growth conditions and the bacterial species of the chlorosomes, the structure as a whole is probably best described as a plastic crystal, where the *syn-anti* self-assembly provides a cage with near tubular symmetry and with the monomeric building blocks subject to restrained molecular rotational dynamics in the plane of the macrocycles. This may represent a commonality that is preserved across different chlorosomes to sustain their light harvesting function.^17^

Due to the close packing of the bacteriochlorophyll molecules, the excited states responsible for light absorption are strongly coupled.^18^ At the same time the structural disorder and interaction between different molecules leads to variation in the couplings and excitation energies of the individual bacteriochlorophyll molecules. The competition between coupling and disorder leads to the formation of exciton states delocalized over numerous molecules. The dynamic nature of the disorder gives rise to efficient long-range transfer of the energy absorbed by the antenna as revealed by pump–probe^1^ and two-dimensional electronic spectroscopy,^7^ and it determines the optical properties of the chlorosomes. For understanding these properties it is, thus, crucial to understand these exciton states and how the localization that inevitably arises from the static heterogeneity is overcome by the dynamic heterogeneity in the plastic crystalline chlorosome self-assembly for rapid redistribution of exciton density and energy transfer.^17^

At an early stage, theoretical descriptions have been developed starting with idealized models considering tubes with a fixed crystalline structure.^18,19^ While this allowed for basic understanding of the spectral features, the extreme exciton delocalization of individual exciton states is unphysical, as localization caused by the presence of the disorder is not included.^20^ Such phenomenological models do not reveal the microscopic origin of the disorder that governs spectral broadening and defines the observed line widths. A hybrid approach which combines molecular dynamics (MD) simulations and time-dependent density functional theory (TD-DFT) for a tubular structure with 1200 bacteriochlorophylls^21^ allowed for an insight into how the presence of dynamic disorder in the system enhances ultrafast exciton transfer. Recently, an exciton model was developed based on the MD simulations of a triple tube model system which contains more than 25000 bacteriochlorophyll molecules.^22^ The latter study revealed the presence of hydrogen bonding patterns in the structure and the presence of a persistent rotational mode with a frequency comparable to the oscillation observed in time-dependent studies^23^ that was assigned to the presence of vibrational coherence. This exciton model explicitly included disorder in the excitonic coupling while neglecting the presence of disorder in the excitation energies of the chromophores. It allowed the study of the ultrafast energy transfer within and between the concentric cylinders.^20^ In this work, we will extend the presented theoretical studies^20,22^ by including disorder in the excitation energies, which is important for a detailed description of how the heterogeneity in the local structure impacts the energy landscape. Furthermore, we will provide simulation of the linear and nonlinear optical spectra based on the microscopic model. Connection of our results to experimental observations^7,8,15^ allows us to understand how molecular scale disorder affects spectroscopic properties in the system.

The remainder of this paper is organized as follows. First, in the “Methods” section we will summarize the information on the structure and MD simulations. Furthermore, the exciton model will be described and the quantum chemical parametrization used for determining the disorder in the excitation energies. Then the method for simulating the optical spectra will be summarized, and the methods for performing the analysis of the structure and exciton states will be summarized. In the “Results” section, we will present the linear absorption spectra and analyze the origin of the disorder with a focus on the role of hydrogen bonding, angular disorder, and *syn-anti* motifs. We will present the calculated two-dimensional electronic spectra (2DES) of a single-tube subsystem constructed by truncating the final Hamiltonian of the complete system. The simulated spectra will be compared with the experimental observations and the spectral features will be discussed. In particular, we will connect with the calculated quantum delocalization of the states dominating the spectrum. Finally, we will summarize the findings and present our conclusions.

## Methods

In this section, we will describe the multiscale modeling procedure that we used to simulate the linear and nonlinear optical response of the *Q*_*y*_-band of chlorosomes. The workflow is already used for prediction of spectroscopic signatures of different systems, starting from the atomistic simulations.^21,24−26^ As an initial model of chlorosomes we chose the atomistic structure of the three tube complex. Dynamics and disorder effects, coming from the structural fluctuations, are obtained based on the all-atom molecular dynamics simulations. These two initial steps were already described in detail previously,^22,25^ and only a brief summary will be given below. The energy landscape of the *Q*_*y*_-band is represented with the Frenkel exciton model, that was parametrized to include described effects of structural inhomogeneities. The description of the quantum dynamics in the system and its optical response was obtained by the Numerical Integration of the Scrödinger Equation (NISE) approach.^27,28^ Comparison of our results with the observations from optical experiments allows us to validate the quality of the initial model and provide insight on the effects that structural disorder has on the character of exciton states and optical properties. In the following subsections, each of these steps will be described in more detail.

### Initial Structural Model

For an initial structure we will use the proposed model of the concentric cylindrical aggregates built from curved sheets of *syn-anti* stacks.^12^ This model is in agreement with previous cryo-EM,^11^ solid state NMR,^12^ and single-molecule optical^13^ and microscopy experiments.^29^ The sheet structure is defined by the lattice parameters (*a*, *b*, γ) which describe the local structure. The radius *R* and the chiral angle δ are geometrical parameters which are defined by the way these sheets are rolled to form the tubes of different size and chirality.^25^ All these parameters will affect the positions of molecules and their transition dipole moments within the cylinder which will dictate the optical response of the system.^30^ In this work, we will use previously defined values for these parameters.^25^ The lattice parameters were (*a*, *b*, γ) = (1.48 nm, 0.98 nm, 124.3°), and the chiral angle was δ = 49.6°. The initial radii of the three concentric tubes used were *R* = 5.2, 7.3, and 9.5 nm, respectively. The length of each tube was set to 120 nm. The whole system has 27675 molecules, which allows for a statistical characterization of the structural disorder. A schematic representation of the main structural motifs: *syn-anti* alternating parallel stacks and hydrogen bonded pairs which connect stacks is shown in Figure 1.

Simulations of the 2DES spectra have high computational cost due to the scaling as *N*^3^ with the number of molecules.^31^ To overcome this issue, we defined a representative smaller subsystem on which this calculation is feasible. The smaller system consists of a part of the middle tube that contains 2639 molecules and has length and radius of 35 and 7.5 nm, respectively. We confirmed that this system is a valid representation of the system of interest, since the qualitative behavior in the fluctuations of energy parameters did not vary significantly compared to those observed for the full three tubes.

### Molecular Dynamics Simulations

The initial three-tube complex was equilibrated for 1 ns at 300 K with an unconstrained all-atom molecular dynamics simulation in the canonical ensemble, using the OPLS-AA force field,^32^ as implemented in GROMACS.^33^ Since we are interested in ultrafast dynamics in the system, a 10 ps long production run trajectory was generated, where molecular dynamics frames were stored every 20 fs. A detailed description of the equilibration procedure and molecular dynamics parameters is given in ref (25).

### Model Hamiltonian

With the assumption that only *Q*_*y*_ excitation of the BChl *c* molecule is of interest, we will adopt the framework of a single excitation Frenkel exciton Hamiltonian, which is here given in the site basis:^34^
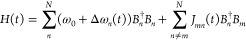
1Here,  and *B*_*n*_ are the creation and annihilation operators describing an excitation on the *n*th BChl *c* molecule. *N* is the total number of molecules. The first term represents the transition energy of the monomer evaluated as a sum of the energy of the *Q*_*y*_ transition of BChl *c* in vacuum ω_0_ and the energy shift that arises due to interaction with the local environment, Δω_*n*_(*t*). The second term gives the excitonic couplings *J*_*mn*_ between the different BChl *c* molecules. For ω_0_ we chose the value of 15390 cm^–1^, which corresponds to the energy of the *Q*_*y*_ transition of the BChl *c* monomer in methanol.^35^ A schematic description of the Bchl *c* that is surrounded with other chromophores within the aggregate is shown in Figure 1b). The energy shifts are calculated as the Coulomb intermolecular interaction:^36^
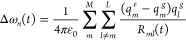
2Here, *n* labels the specific BChl *c* molecule. *m* runs over all *M* atoms in this molecule, and *l* runs over *L* atoms on the surrounding molecules.  and  are partial charges of the atoms of the molecule of interest that reflect its excited and ground state properties.  are the ground state charges on the atoms of the molecules in the local surroundings. The distance between the atoms is given by *R*_*ml*_(*t*). In this way, we include the difference in the effects that electrostatic potentials of the local environment have on the ground, compared to the excited states, of the molecules in the aggregate. The partial charges were determined from quantum chemistry calculations as described in the section Quantum Chemical Parametrization. A 20 Å cutoff excluding molecules with a Mg–Mg distance with the central molecules larger than this was used for the electrostatic interactions. This is identical to the cutoff used in previous calculations of the same type on other systems.^24,37^ The dependence on the local structure and the dynamic fluctuations of this term lead to what is referred to as diagonal disorder in the Hamiltonian.

We describe the excitonic coupling in eq 1 using the point dipole approximation similarly as in the previous chlorosome study:^25^
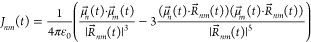
3Here,  is the transition-dipole moment vector for molecule *n*, and ) is the distance vector between the centers of the two molecules, which we define with the position of the magnesium atoms. No explicit dielectric screening was included following the protocol of ref (25). We used a transition-dipole moment of 5.48 D in accordance with findings for BChl *c* monomers in methanol solution,^35^ which may implicitly account for some of the dielectric screening in the couplings. The direction of the transition-dipole moment was defined to be along the vector between the nitrogen atoms conventionally named *N*_*A*_ and *N*_*C*_ as shown in Figure 1. This dipole is identical to that used in previous studies on static structures.^12,13,38^ The dispersion of molecular orientations and distances within the aggregates will lead to a dynamic distribution in the excitonic couplings, also denoted as off-diagonal disorder.^24,39^ For characterization of this disorder we will use the coupling strength which is the signed sum over all pairwise interactions involving a specific BChl *c* molecule:
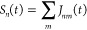
4*S*_*n*_(*t*) gives a single fluctuating quantity per molecule which makes analysis simpler. Dispersion in the exciton couplings will influence the width and the position of the excitonic band.^24,40^ We note that the conditions for the validity of the point dipole coupling approximation is possibly not fully met as the chromophores are closely packed. Applying a more accurate extended dipole^24^ or TrESP^41^ coupling approach may improve the current description. Inclusion of such a coupling scheme for the description of excitonic dynamics is computationally demanding, especially for large systems such as chlorosomes and presents a challenge for future studies. As the present coupling model shows good agreement with experiments we do not expect improving the coupling model to affect the conclusions of this paper.

### Quantum Chemical Parametrization

To determine the charges needed to evaluate the transition energy shift described in eq 2 we modeled the ground and the excited states of the Bchl *c* monomer. All calculations were performed as implemented in the ORCA 4.2.1. software.^42^ The initial structure of a BChl *c* molecule was taken from the MD trajectory, and its geometry was optimized using density functional theory (DFT) with the hybrid exchange-correlation PBE0 functional^43^ and def2-SVP^44^ basis set. To describe vertically excited electronic states for the optimized geometry, we performed the (TD-DFT) calculations using the range-separated ωB97x^45^ functional with split-basis approach where we used the def2-SVP^44^ basis set for all carbon and hydrogen atoms. Larger basis sets were used to to describe nitrogen and oxygen (def2-TZVP) and magnesium atoms (def2-TZVPD).^44^ Geometry optimization and calculation of vertical excitations were performed using the RIJCOSX approximation for Couloumb and exchange integrals with auxiliary def/J basis set.^42^ The TDDFT calculations yielded an excitation energy of the *Q*_*y*_ transition of 15926 cm^–1^ and a transition dipole moment of 3.83 D.

The partial charges of the atoms in the ground and first excited state are determined using the CHELPG method.^46^ Within this scheme the charges of the atoms are evaluated to reproduce the electrostatic potential generated by the full electronic density. The procedure is constrained to reproduce conservation of charge of the molecule and we used a grid of 2500 points per atom for fitting the electrostatic potential. The calculation of the partial charges was performed using the Multiwfn software^47^ based on the electron densities obtained from the previously described DFT and TD-DFT calculations. The resulting charges used for evaluation of the energy shifts, as described with eq 2, are given in the Supporting Information. In this approach, the partial charges are, thus, approximated to be independent of the internal molecular vibrations in accordance with previous models^24,36^ of similar systems.

### Spectral Simulations

The essence of the NISE method^27,28^ is to use the explicit solution of the time-dependent Schrödinger equation:
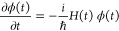
5for the time-dependent system Hamiltonian as given in eq 1. Propagation of the wave function is evaluated by dividing time into small intervals and using solutions of the approximate time-independent Hamiltonian calculated for every interval. For one such time step of duration Δ*t*, we thus have
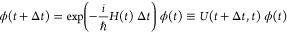
6This defines the time-evolution operator *U*(*t* + Δ*t*, *t*). The time-dependence in the Hamiltonian is here parametric as the Hamiltonian is different in each time step due to the fluctuation of the environment. For longer time intervals the time-evolution is then defined by successive time-ordered multiplication of time-evolution operators:
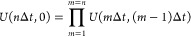
7Different types of spectroscopic observables can then be obtained from response function expressions^27,48^ using the NISE_2017 program.^28,49^ The NISE approach results in unitary time-evolution operators and thermalization to the infinite temperature equilibrium, but it has been shown to describe both absorption spectra and short waiting-time 2DES spectra accurately.^50^ Assuming that we are in the impulsive limit, the expression for the linear absorption is given by
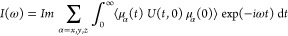
8Here, μ_α_(*t*) is a vector with the Cartesian-components of the transition dipole moments of the BChl *c* molecules at time *t* given by α. For the practical simulations, the integral of eq 8 is evaluated as a Fourier transform on a time grid with values calculated at a time step of Δ*t* = 4 fs to achieve the needed width of the spectral window. Here we used a coherence time of 128 fs, which sets the time at which the integral was truncated. The calculated spectral lines were convoluted with Lorentzian and Gaussian apodization functions.^51^ This is achieved by multiplying the time-domain response functions with an exponential, , and a Gaussian function, , respectively. This accounts for the effects of homogeneous and inhomogeneous broadening arising due to the effects of mesoscopic scale disorder.^52^ The time scale chosen to describe effects of homogeneous broadening was fixed to τ_homo_ = 300 fs. The relevant time scale of the inhomogeneous broadening corresponds to the lifetime of τ_inh_ = 166 fs, in line with the estimates from hole burning studies.^53^ Performing the convolution results in smoother spectra, while spectral positions and line widths do not change significantly, as long as lifetimes of τ_homo_ and τ_inhomo_ are significantly slower than the dephasing time resulting from the molecular scale energy disorder included already in eq 8 (about 66 fs).

Equivalently, for the two-dimensional electronic spectra at the *t*_2_ = 0 ps time was calculated using the corresponding response functions,^27^ but for the extended coherence times up to 192 fs.^26^ Absolute third-order nonlinear optical response is evaluated as a sum of rephasing and nonrephasing contributions.^48,51^ The spectra were obtained by averaging over 10 different configurations along the molecular dynamics trajectory. Consistent with the calculations of the linear absorption, we accounted for the effects of the mesoscale disorder on the spectral lines by convoluting simulated spectral lines with Lorentzian and Gaussian apodization functions.^51^ In this way, the results can be compared directly with the experimental spectra, which are performed on the chlorosomal ensembles.^7,23,54^Figure S3 shows the calculated 2D spectrum without the mesoscale disorder.

## Results

**Figure 2:**
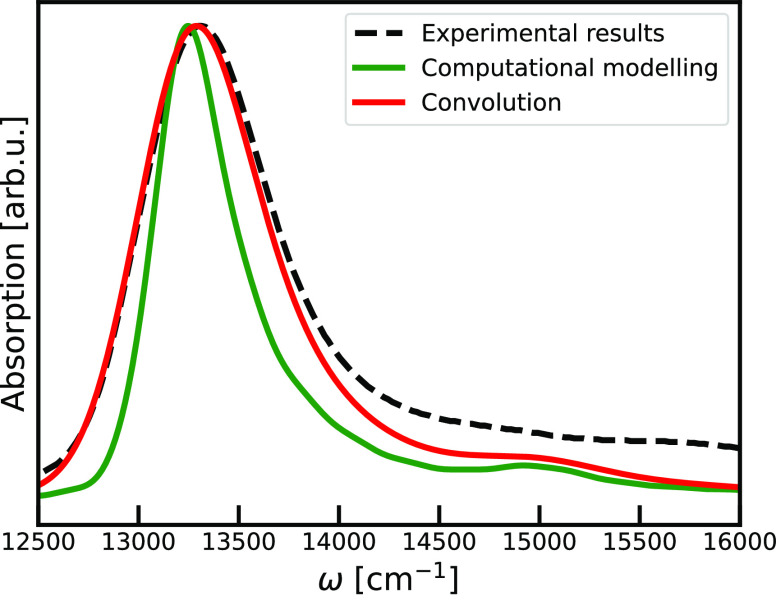
Comparison of the linear absorption spectra of the three-tube model (green line) with the total spectra which represents convolution of the simulated spectra with the larger scale disorder contribution (red line) and experimental spectra of chlorosomes from wild-type green sulfur bacteria *Chlorobaculum tepidium* (black dashed line) (unpublished results).

The simulated absorption spectrum of the Q_*y*_-band of the triple tube model system is shown in Figure 2. This spectrum was shifted by 1350 cm^–1^ to the red to match the position of the experimental absorption maximum. The bare simulated spectrum is narrower than the experimental one, since it only accounts for disorder at the microscopic level, where the experimental spectrum also reflects presence of the mesoscopic scale disorder coming from the variation in tube sizes (predominately difference in radii) and orientations.^7,16^ To account for this effect, that leads to additional inhomogeneous linebroadening, we convoluted modeled spectrum with the Gaussian band, with a standard deviation of σ = 200 cm^–1^ to achieve better agreement to the experiment. Comparing with the previous simulations of ref (25) and by neglecting the diagonal disorder (not shown), we found that the molecular scale spectral broadening almost exclusive arises from the diagonal disorder.

Our microscopic model predicts an absorption band with a full-width-half-maximum (fwhm) of 495 cm^–1^, while the experimental line width is 800 cm^–1^. Adding effects of the inhomogeneous broadening to the simulated spectra leads to the absorption band with the fwhm of 765 cm^–1^, which is in accordance with the experimental observation. Overall, the skewed shape of the simulated spectra is in a good agreement with the experimental line shape. The lower intensity on the blue side of the simulated spectrum may originate from the *Q*_*x*_-band and tails of the Soret band at higher frequencies, which are not accounted for in the present model. Additional contributions to the spectral intensity and broadening could arise due to the coupling of the excitonic states to intramolecular vibrations^55−57^ or to the charge transfer states.^58^ Since these effects are neglected in the current model, they could be a cause for the discrepancy between the simulated and the experimental spectra in the high energy region. This effect must be small as mixing with charge transfer states would lead to fast exciton quenching, which is not observed experimentally.

**Figure 3:**
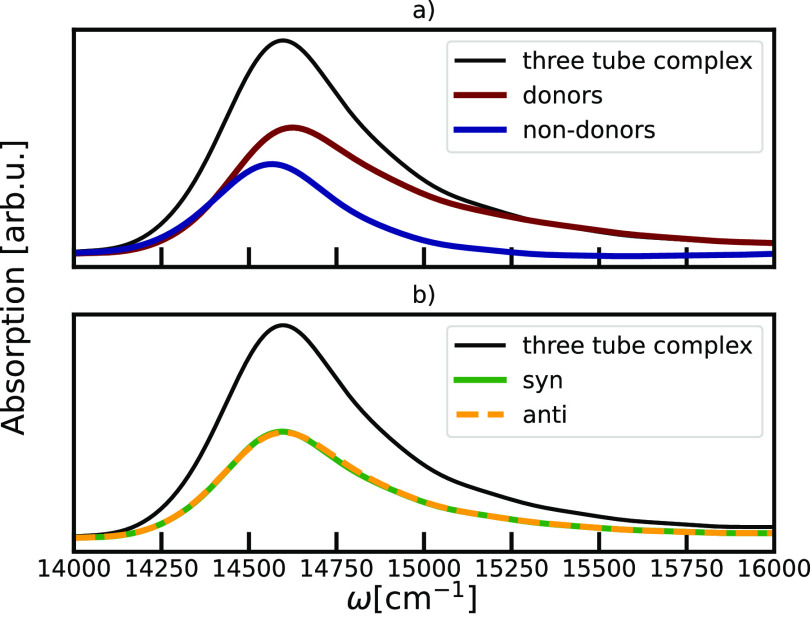
Linear absorption spectra projected on subensembles of molecules depending on their structural behavior: a) with the respect to their role as the donors of the hydrogen bond, b) the type of monomer in the stack (*syn* or *anti* configuration).

In Figure 3, we show the analysis of the effects of the molecular scale of disorder on the absorption spectra. Through projection,^59^ the spectral response was decomposed to contributions coming from chromophores donating a hydrogen bond and ones that do not have that role. Well in line with the previous analysis of the MD simulations,^22^ we found that ∼70% of the BChl *c* molecules are hydrogen bond donors, while ∼30% are not. We will refer to these groups as donor and non-donor molecules. Interestingly, the 70:30 distribution is comparable to the subcomponent ratio previously reported from NMR studies.^12,60^ The spectra of these two components are quite distinct, as shown in Figure 3 a) where absorption coming from donors is peaking at a higher frequency and is completely dominating the high energy tail above 15000 cm^–1^. The distance between the peak maxima coming from two components is ∼40 cm^–1^. This observation is well in line with the experimental report of the two components in the spectrum of individual chlorosomes.^61^ The difference in hydrogen bonding leads to inhomogeneous broadening in the spectrum. Changes of the absorbance of BChl molecules caused by the changes in the number of the hydrogen bonds were experimentally observed in, for example, LH2^62^ and LH1 complexes^63^ of purple bacteria. In contrast, as shown in Figure 3b we do not observe any significant difference in the spectral response of the *syn*- and *anti*-configurations of the molecules, which show only small differences in their energy disorder.^64^ We note that such distinction can still arise from intramolecular vibrations as explicitly investigated in other light-harvesting complexes.^55−57^ Such effects were not included in our model as the calculations would be prohibitively expensive due to the give system size. It has been shown that local effects coming from intramolecular vibrations are strongly suppressed in systems with large exciton delocalization^65,66^ as we found here, and we thus expect the effects of intramolecular vibrations to be small. Molecules in the *syn*- or *anti*-configurations do not exchange, and it was found that hydrogen bond exchange is too slow to happen in the present simulations.^22^

**Figure 4:**
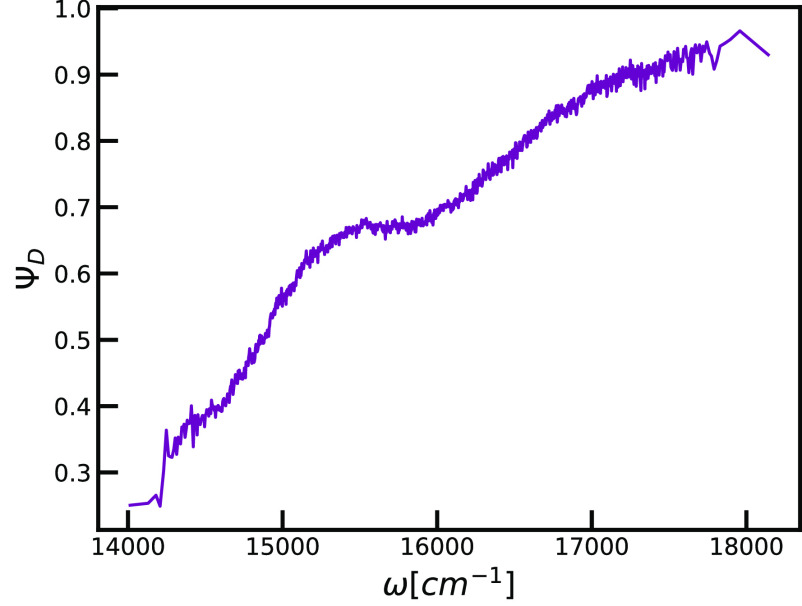
Donor character of the exciton states as defined by eq 9 plotted as a function of the exciton energy.

To quantify how hydrogen bond donor compared to non-donor molecules contribute to the exciton states, in a given energy region, we define the donor character, parameter that can take a value between 0 and 1:
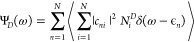
9where  is 0 if molecule *i* is not donating, and 1 if it is donating a hydrogen bond. *c*_*ni*_ is an expansion coefficient, a number between 0 and 1, which shows how molecular states contribute to the exciton state of interest. In Figure 4, we show how the donor character Ψ_*D*_ changes within the exciton band. Ψ_*D*_ reaches its limiting values on the edges of the band, revealing that the main contribution to low-lying exciton states comes from non-donor molecules. On the contrary, donor molecules predominantly characterize states in the high energy side of the band. These findings are in agreement with the observations in the calculated absorption spectra and the contributions of two spectral components shown in Figure 3a.

**Figure 5:**
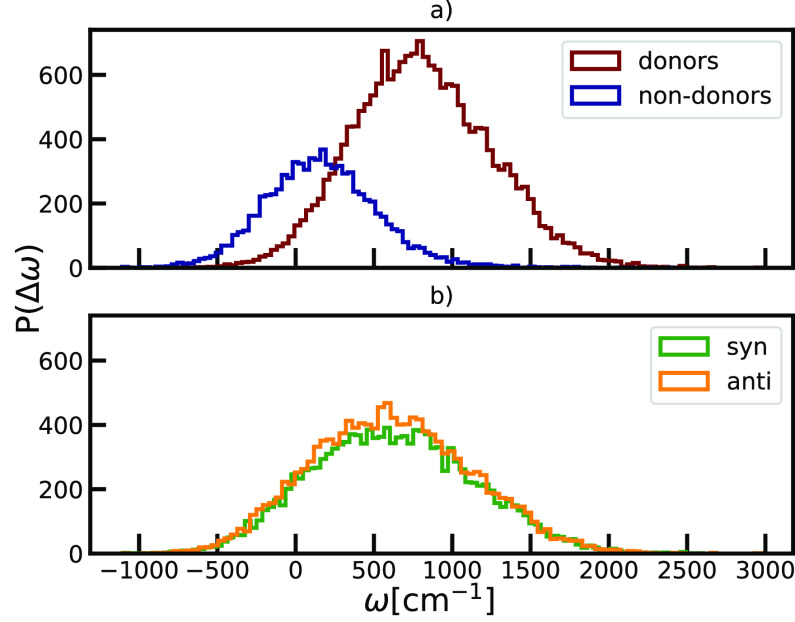
Comparison of the distributions of the disorder in the transition energies depending on the role of the molecules as a) hydrogen bond donors or not and b) type of building blocks within the unit cells: *syn* or *anti*.

In Figure 5, we first show the distributions of transition energies in the system also known as diagonal disorder. We observe the trend of the transition energies shifting toward the higher values compared to the *Q*_*y*_ transition of BChl *c* in vacuum. Hydrogen bonding greatly affects the heights of energy gaps of the *Q*_*y*_ transition, which is in contrast to the effect coming from structural differences between *syn*- and *anti*-monomers. We connect this observation to the difference in the strength of the interactions. Hydrogen bonding, as a very strong noncovalent interaction, affects the packing and the charge distribution within the local environment significantly. On the other hand, different orientations of the farnesyl tail with the respect to planar porphyrin ring, which is an intramolecular effect used to assign the type of monomer within the *syn*/*anti*-stacks, manifest as a more subtle effect. Enhanced structural stabilization of the ground states of donors leads to more prominent blue-shift than observed for non-donor molecules. The observed behavior is understood based on the structural analysis that is included in the Figure S1.

**Figure 6:**
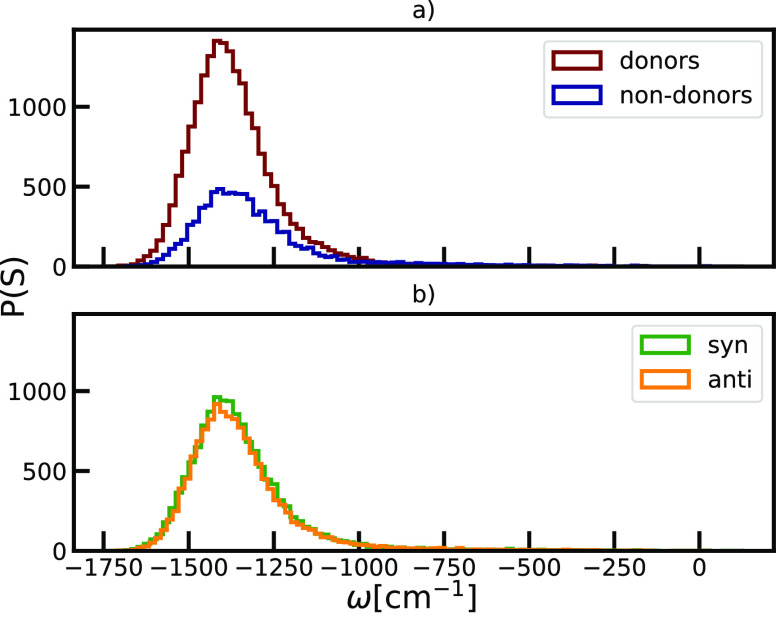
Comparison of the distributions of the disorder in the excitonic coupling, described with the coupling strength parameter, depending on the role of the molecules as a) donors of the hydrogen bond and b) type of building blocks within the unit cells: *syn* or *anti*.

In Figure 6 we present an analysis of the distribution of the coupling strength. The skewness of the distributions, influenced by the cylindrical geometry of the system which promotes long-range interactions, can in part explain the observed skewness of the overall absorption spectra. The coupling strength is negative, leading to the spectral red-shift, as commonly known from J-aggregates.^67^ Molecules with a role of donors of hydrogen bonds, on average experience stronger effective excitonic coupling compared to the non-donor molecules. The exciton coupling between a given pair of molecules is determined based on the Mg–Mg distance and the angle Θ in the transition-dipole coupling scheme shown in eq 3. Hence, in agreement with the structural analysis, the role that molecules have in hydrogen bonding significantly affects the exciton coupling, giving larger values for donor molecules which have a more prominent overlap between the porphyrin rings. In contrast, the coupling strength distributions for *syn*- and *anti*-conformations are identical. Statistical information, which provides more insight in the differences between the energy shifts and strengths of the disorder distributions, is given in Table 1.

**Table 1:** Characterization of the Distributions of the Diagonal and off-Diagonal Disorder for Different Structural Inhomogeneities: Comparison between *syn* and *anti* (Top) and between Donor and Non-Donor Molecules (Bottom)

Comparison between *syn* and *anti* Chromophores		
	⟨Δω⟩(σ) (cm^–1^)	⟨*S*⟩(σ) (cm^–1^)
*syn*	620 (514)	–1348 (166)
*anti*	611 (510)	–1347 (165)

Hydrogen Bonding: Comparison between Donor and Non-Donor Molecules		
	⟨Δω⟩(σ) (cm^–1^)	⟨*S*⟩(σ) (cm^–1^)
donor	807 (442)	–1365 (142)
non-donor	165 (363)	–1307 (205)

**Figure 7:**
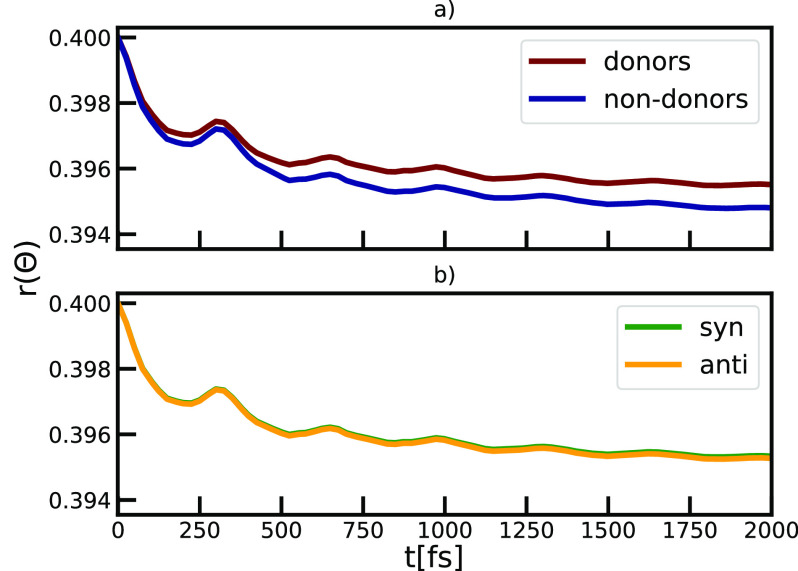
Orientational correlation function obtained for different subensembles, depending on their roles in a) hydrogen bonding and b) *syn*- or *anti*-stacking.

Up to this point, we characterized the static disorder, which comes from the structural inhomogeneities that are present in the system. Fluctuations in the positions of the atoms, as described with molecular dynamics simulation, will cause energy fluctuations also known as dynamic disorder. We quantified the dynamics of these effects through calculations of the time-dependent autocorrelation functions of parameters of interest. Ultrafast rotation of the BChl *c* molecules in chlorosomes was previously observed in the molecular dynamics simulation,^22^ and it is proposed that this mode can drive ultrafast exciton transfer observed in the chlorosome.^20^ We will describe this rotational degree of freedom by evaluating the renormalized orientation correlation function:^68,69^
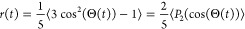
10Here, Θ(*t*) is the angle between the transition-dipole moment vector of a BChl molecule (Figure S1) at two different times separated by the time *t*. *P*_2_ is the second order Legendre polynomial. The ^2^/_5_ factor used here connects with the spectrally relevant anisotropy parameter,^68,69^ which describes the perfect correlation with 0.4 instead of 1. Rotation of the chromophores affects the mutual alignment of the transition dipole moments with respect to other molecules leading to variations in the excitonic couplings and spectral response. The calculated orientation correlation functions are shown in Figure 7. Overall, the orientational correlation functions decay to about 0.395. This corresponds to a cone angle of rotation of 9°.^69^ A low-frequency oscillation is observed in all orientational correlation functions, demonstrating the twist of the flat ring structures is an underdamped type of motion, which may be picked up in time-resolved anisotropy experiments. The rotational behavior is identical for *syn* and *anti* molecules. However, we observe more restricted motion of donor molecules compared to non-donors. This finding is in agreement with the structural analysis showing enhanced stabilization of the ground state of the donor compared to non-donor molecules, given previously.

**Figure 8:**
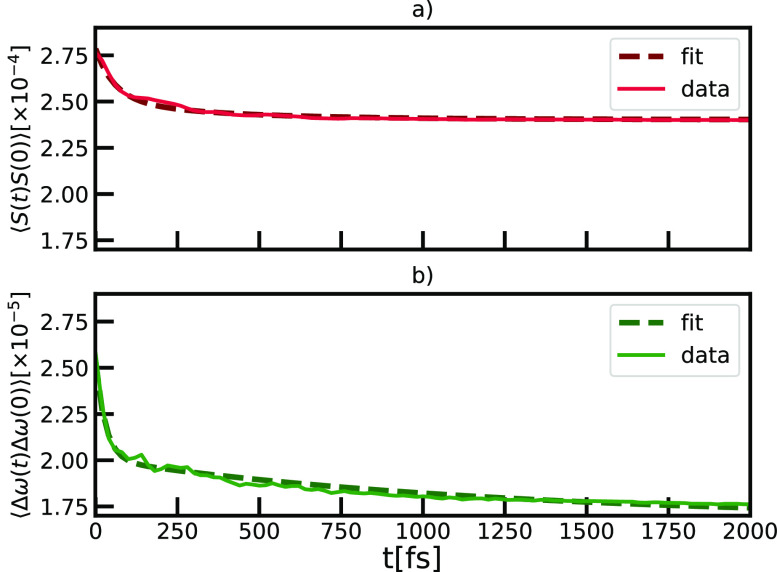
Comparison of the autocorrelation functions for the different parameters of the system: a) diagonal disorder and b) off-diagonal disorder.

The energy fluctuation dynamics dictate the spectral response and define the position and the line shape of the absorption band.^70^ We will characterize these fluctuations by evaluating temporal autocorrelation functions:
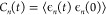
11Here, ϵ_*n*_ can take the values of the transition energies or coupling strengths. Calculated autocorrelation functions were fitted to the multiexponential function:
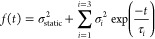
12The fitting coefficients which give the strengths σ_*i*_ and time scales τ_*i*_ that describe dynamical processes in the system, are presented in Table 2. Calculated autocorrelation functions of the fluctuations in the transition energies and the coupling strengths, alongside with the corresponding multiexponential fits, are shown in Figure 8.

**Table 2:** Parameters for Fits of the Autocorrelation Functions with eq 12 for Diagonal Disorder and for off-Diagonal Disorder^a^

parameter	diagonal disorder	off-diagonal disorder
σ_static_ (cm^–1^)	410	155
σ_1_ (cm^–1^)	230	45
σ_2_ (cm^–1^)		30
σ_3_ (cm^–1^)	185	30
τ_1_ (fs)	1200	400
τ_2_ (fs)		100
τ_3_ (fs)	50	50

a The slowest time scale has index 1, and the fastest has index 3.

Based on the information about different dynamical contributions, summarized in Table 2, we show that autocorrelation functions of the disorder in the transition energies can be fitted with two exponential contributions, while for the complete fit of the correlation functions of the coupling strength additional component is needed. With this procedure, we are able to estimate the time scales τ_*i*_, of different dynamical processes, which arise from the structural fluctuations included in our microscopic model that dictate the complex dynamics in chlorosome. We also quantified different amounts of disorder σ_*i*_ corresponding to observed dynamical processes alongside the static disorder contribution, which is evaluated as a cutoff. We note that complex multiexponential dynamics in chlorosomes was also observed in the 2DES experiments.^23^

**Figure 9:**
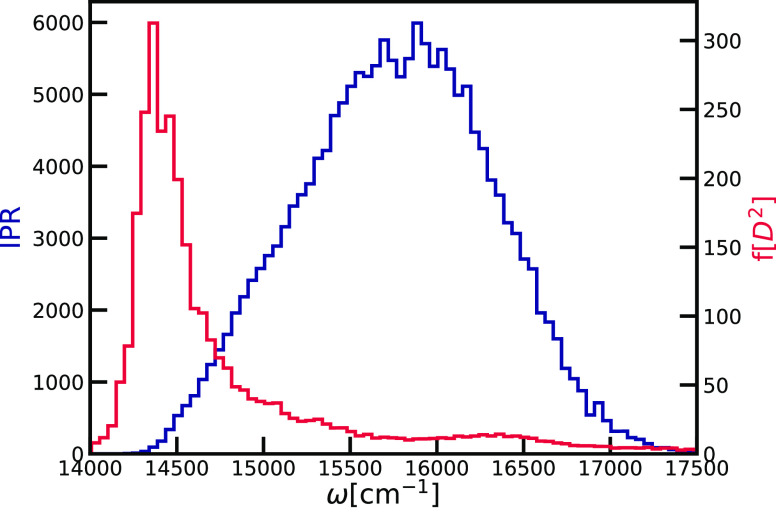
Characterization of the excitonic states: inverse participation ratio (blue histogram) and oscillator strength f (red histogram) plotted against the density of states.

The inverse participation ratio (IPR)^71^ provides a measure on the number of molecules that contribute to the excited states in a specific spectroscopic region:
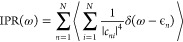
13where *c*_*ni*_ is the wave function coefficient that describes the contribution of the molecule *i* to the exciton state *n*. ϵ_*n*_ is the energy of state *n*, and ω is the frequency, for which the inverse participation ratio is calculated. The transition-dipole moment of the excitonic states is given as a weighted sum of all molecular transition dipole moments :
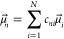
14In Figure 9, we show how the IPR changes within the excitonic band. States on the edge of the exciton band will be the most affected by disorder which results in their localization. Non-donor molecules contribute more to these states as they are lower in energy. We further show the oscillator strength  as a function of exciton energy. Representation of the dependence of the oscillator strength, as evaluated for the initial snapshot, is given in Figure 9. All the oscillator strength is distributed over the low-energy region of the exciton band, which is a known characteristic of the J-aggregates. The disorder causes the redistribution of the oscillatory strength over more states than it is expected for a perfect homogeneous aggregate. We estimate that in the optically active regime of the exciton band there is still prominent delocalization on over hundreds of chromophores. This is clear from the low-energy part of the exciton band, which is shown in Figure 9. Within the band, with increasing energy of states, the value of the IPR rises until it reaches the maximum value in the middle of the band, after which it decreases again, as shown in Figure 9. This localization–delocalization crossover^72^ characterized by changes of the IPR with respect to the density of states. This observation is a consequence of higher sensitivity of the states on the edge of the bend to the presence of the disorder.

Another way of characterizing the delocalization of the exciton states is through comparison of the magnitude of the transition dipole moments of the excitonic states with the magnitude of the molecular transition dipole moments. This estimate of the delocalization is only relevant for the optically active part of the band. In homogeneous aggregates, where all transition dipole moments have the same orientations and magnitudes, all molecular transition dipole moments will be excited in phase leading to the complete delocalization of the exciton states over all N molecules.^73^ The transition dipole moment of the described exciton state, also called the super-radiant state, will be a weighted sum of all molecular transition dipole moments given as ,^73^ where N* is the effective number of sites involved in the delocalized state.

**Figure 10:**
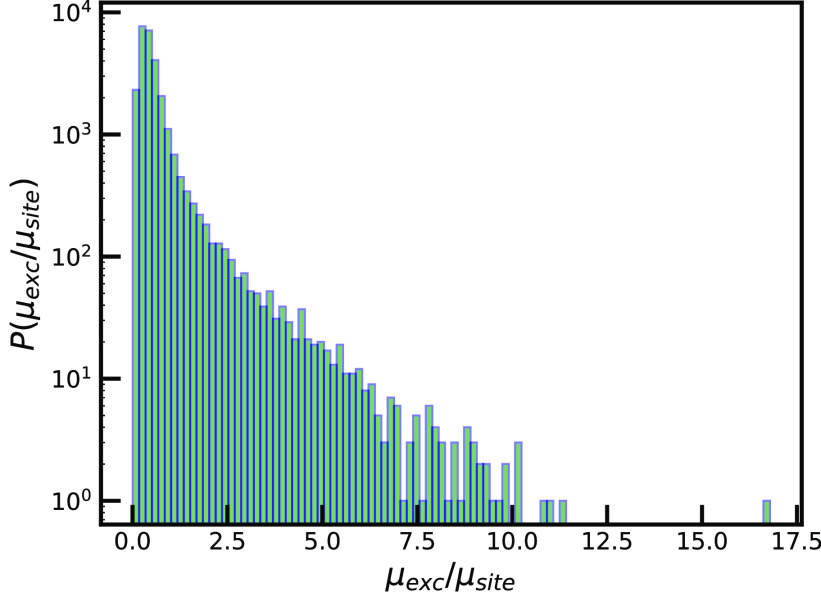
Log-scale histogram of the magnitude of the exciton transition dipole moment in units of the molecular transition dipole moment.

The histogram of transition dipole moments of the exciton states of chlorosomes is presented in Figure 10. We report that most of the states are dark, with a transition dipole moment close to zero. We observe a couple of super-radiant states, for which the ratio of μ_*exc*_/μ_*site*_ is greater than 10. These states dominate the absorption spectra, since their optical response depends on the oscillator strength that is given as a square of the transition dipole moment. Comparing the μ_*exc*_/μ_*site*_ ratio that we found for super-radiant states in the chlorosome with the relation derived for the homogeneos aggregate, we find that these states delocalize over hundreds of molecules. This is in good agreement with the analysis of the IPR that is given above.

### Two-Dimensional Electronic Spectra

Simulations of the 2DES spectrum, require the treatment of double-excited states and are computationally very demanding for large systems.^31,74^ Therefore, it was only feasible to calculate 2DES for a smaller 35 nm long subsystem as defined in the “Methods” section. We verified that the linear spectrum of the subsystem reproduces well the three-tube complex spectrum as shown in Figure S2.

**Figure 11:**
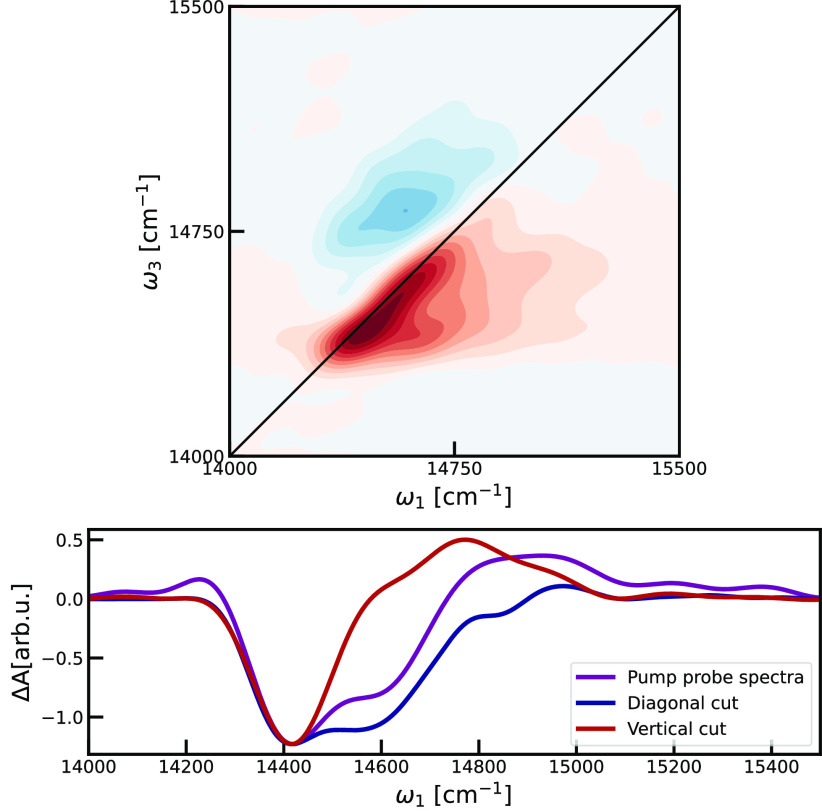
a) 2DES for the *t*_2_ = 0 waiting time and parallel polarization pulse sequence. The spectrum is normalized with respect to the point with maximal absolute intensity, and contour lines are drawn for every 10% of the maximal intensity. Red highlights the bleach signal, while blue shows areas with absorption. b) Corresponding pump–probe spectrum, a vertical slice through the 2DES spectrum at ω_1_ = 14455 cm^–1^, and diagonal slice (ω_1_ = ω_3_).

The calculated parallel polarization 2DES spectrum of the subsystem is shown in Figure 11 at zero waiting time. The general structure of the 2DES spectrum matches the experimental data^7,75^ very well. The spectrum has two main peaks. On the diagonal a bleach peak is elongated in the diagonal direction and fairly narrow in the antidiagonal direction. This peak contains ground state bleach (GSB) and simulated emission (SE) contributions. Above the diagonal a weaker and rounder absorption peak is seen. This peak is originating from excited state absorption (EA). The simulated spectrum does have a bit more structure than the experimental one. This may be attributed to noise arising from the limited simulation time and potential remnants of finite size effects.^38^

To quantify the features in the 2DES spectra the pump–probe spectrum was calculated by integrating over ω_1_. Additionally, we made a vertical slice through the 2D correlation map at ω_1_ = 14455 cm^–1^ and a diagonal ω_1_ = ω_3_ slice. The slices and pump–probe spectrum are shown in Figure 11. The slices are analyzed by evaluating the intensity ratio between the two main peaks (|*I*_*min*_/*I*_*max*_|), the distance between them (Δω), and the spectral full width half-maximum (fwhm) widths of the observed features. The determined values are summarized in Table 3. The ratio between the two peak intensities reflects the contribution of the single compared to double-excitation processes. The distance between the peaks is related to the Pauli exclusion principle preventing two excitations to be present on the same chromophore, and this repulsion energy results in the blue shift of the EA signal compared to the GB/SE ones.^76^ This parameter was evaluated for the vertical slice and the pump–probe spectrum. However, the diagonal slice does not pass through the EA peak, and no peak distance is observed in this slice. We report the values of the blue-shifts between positive and negative peak Δω of ∼350 and ∼550 cm^–1^ for the vertical slice and pump–probe spectra, respectively. These numbers are also relevant for the discussion of the exciton delocalization length that will follow later in the text. Based on the values of the ratio of the positive and negative peak, as given in the Table 3, we see that the contribution of the single-excitations to the spectra is three-times larger compared to processes involving double-excitations. This ratio is larger for the vertical slice, as a consequence of the fact that this spectrum reflects behavior of the specific exciton state in contrast to pump–probe that shows the averaged behavior of the optically active part of the exciton band.

**Table 3:** Parameters Describing Different Transient Absorption Spectra as Shown in Figure 11^a^

parameter	pump probe	diagonal cut	vertical cut
|*I*_*min*_/*I*_*max*_|	0.3		0.4
|Δω_*min* –*max*_| (cm^–1^)	508	554	354
fwhm (cm^–1^)		270	120

a |*I*_*min*_/*I*_*max*_|, absolute value of the intensity ratio between the points of minimal and maximal intensity; |Δω_*min* –*max*_|, absolute value of energy difference between the energy of the points of minimal and maximal intensity; and FWHM of the negative peak which corresponds to the GSB and SE contribution.

The presence of disorder also affects the spectral signatures reflecting the nonlinear response. Here, we observe narrower spectral lines in the vertical compared to the diagonal slice. In this case we compared the width of the negative peaks, which capture the effects of single-excitation processes (GSB/SE). The line width of the negative peak in the vertical slice is dictated by homogeneous broadening. On the other hand, in the diagonal slice we observe all optically active single-excitations which lead to the inhomogeneous broadening effects. Based on the values of fwhm as given in Table 3 of 120 and 270 cm^–1^ for vertical and diagonal slices, respectively, we conclude that the amount of inhomogeneous is two times larger than the homogeneous broadening. Based on the value of the fwhm of the vertical slice, we estimated the homogeneous lifetime of approximately τ_*homo*_ ≈ 300 fs

### 2D Spectroscopy and Exciton Delocalization

Analysis of the time-resolved spectral response is often used to evaluate the delocalization of the excitonic states.^77^ A relation that connects the exciton delocalization length (EDL) to the parameters estimated from the 2D spectra has been developed for linear aggregates:^76^
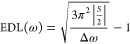
15EDL gives the estimate of the number of molecules which contribute to the bright exciton state and depends on the ratio between the effective coupling strength  and the blue-shift of the ESA peak compared to the GSB/SE peak, Δω. Based on the information from pump–probe and the vertical spectral slice, discussed previously, one can estimate EDL averaged over the exciton band or for a specific exciton energy, respectively. Using this approach to extract the information from the 2D spectroscopy measurements of chlorosomes^77^ yielded the value of EDL ≈ 6 at room temperature. Combining the average value of the coupling strength of |*S*| = 1350 cm^–1^ with the estimates for Δω of 550 and 350 cm^–1^ gives values of EDL of ≈5 and ≈6 for pump–probe and vertical slice, respectively. Calculated values are in a good agreement with the previous report.^77^

However, our analysis of the inverse participation ratio and the super-radiance in chlorosomes shows that exciton states are very robust in the presence of disorder and delocalized on over ≈200 chromophores. Combing this value with eq 15 would require unrealistically high values of the effective exciton coupling  ≈ 1300Δω ≈ 455000 cm^–1^ to reproduce the experimentally observed energy shift of the excited state absorption. With this comparison, we show that using eq 15 in combination with the estimates from time-dependent studies significantly underestimates the delocalization length in chlorosomes. The relation given in eq 15 was derived for linear aggregates,^76^ and we find that it cannot properly describe behavior of the exciton states that emerge in large cylindrical systems like chlorosomes. Higher dimensionality of the system and the presence of curvature lead to emergence of the very delocalized exciton states that are robust to the presence of the molecular disorder.^74^ The prominent delocalization that we find for the exciton states allows for the very efficient exciton transfer observed experimentally.^7,8^

## Discussion and Conclusions

In this paper, we combined a microscopic model of chlorosomes with quantum-classical simulations to obtain a detailed description of linear and nonlinear optical properties of these highly efficient light-harvesting systems. Emerging exciton states are described within the Frenkel exciton model, that is constructed combining parameters from molecular dynamics simulations^25^ and quantum chemical calculations to include effects of disorder in the transition energies extending the previous study which includes fluctuations in the couplings.^20^ The spectral simulations reveal the microscopic mechanism through which molecular scale disorder dictates spectral features as the (in)homogeneous line widths, spectral skewness, and the excited state absorption.

Our microscopic model combined with an addition of mesoscale disorder recovers the experimental absorption spectra very well. Detailed analysis of local structural patterns, shows that *syn* and *anti* molecules cannot be distinguished within the band, and therefore the presence of this motif does not contribute to the spectral broadening. In contrast, the different roles of BChl *c* molecules in hydrogen bonding contribute significantly to the inhomogeneous broadening of the overall spectra. We found that molecules with a role of a hydrogen bond donors absorb at higher frequencies compared to non-donor molecules. The hydrogen bond dynamics is essentially “frozen” on the spectroscopic time scale, and therefore the contribution of hydrogen bonding is fully static. Furthermore, the skewness of the linear absorption on the high energy side of the spectrum is directly connected with the non-Gaussian distribution of the excitonic coupling.

We further examined the simulated 2DES spectra at zero waiting time. The simulated spectra including microscopic and mesoscopic disorder reproduce the experimental spectra^7,23^ very well. We recovered the characteristic diagonal elongation of the spectral signal originating from the interplay between homogeneous and inhomogeneous line broadening processes. The distance between the diagonal peak and the excited state absorption peak was found to be 350 cm^–1^ which is in very good agreement with the experiment.^77^ This peak distance is a known marker of the degree of exciton delocalization in linear aggregates.^78^ We compared the delocalization length predicted by our model with the experimentally estimated value.^77^ We conclude that in the optically active region exciton states extended over up to about 200 molecules in contrast to the much shorter delocalization lengths predicted by combining the peak splitting with the relation applicable to linear aggregates.^78^ Our analysis thus demonstrates that this relationship does not hold for tubular aggregates.

The experimentally observed spectra vary strongly depending on the sample preparation procedure and presence of additional higher levels of disorder alongside difference in molecular scale, like different side chains in BChl *c*. This is, for example, reflected in the variation seen in different mutants and for samples grown under different light conditions. The simplicity of our model explains some of the discrepancies between our predictions and experimental results. Additional effects like coupling of exciton states to intramolecular vibrations or presence of charge transfer states, which can be a source of additional broadening, are also neglected in our molecular model. At this point, the inclusion of these degrees of freedom is not computationally feasible to include for the large structures like chlorosomes.

The extremely optimized process of light harvesting and the exciton transfer observed in chlorosomes, are dictated by the dynamics of the exciton states. This study offers the foundation for understanding the molecular mechanism underlying these processes. Future investigation, based on the simulations of the nonlinear response for longer waiting times and explicit modeling of the exciton diffusion, will allow us to unravel how molecular-scale disorder affects ultrafast exciton transfer and shine light on the origin and potential functional role of the experimentally observed coherent beatings.^23^

## Data Availability

The data that support the findings of this study are available from the corresponding author upon reasonable request.

## Supporting Information Available

Methods, structural analysis of local configurations of donor and non-donor molecules, comparison of the linear absorption spectrum, and simulated 2D spectrum of chlorosomes (PDF)
Calculated CHELPG charges for the Bchl *c* molecules (TXT)
