# Supplementary material for: Manifestation of Hydrogen Bonding and Exciton Delocalization on the Absorption and Two-Dimensional Electronic Spectra of Chlorosomes
Source: J Phys Chem B. 2023 Jan 25;127(5):1097–109. doi: 10.1021/acs.jpcb.2c07143 (PMC9923760; doi:10.1021/acs.jpcb.2c07143)
Supplement: Supplementary file 1 — jp2c07143_si_001.pdf [file jp2c07143_si_001.pdf]

Supplementary Information for: Manifestation of Hydrogen Bonding and Exciton Delocalization on the Absorption and Two-dimensional Electronic Spectra of Chlorosomes

Vesna Erić,[†] Xinmeng Li,[‡] Lolita Dsouza,[¶] Sean K. Frehan,[§] Annemarie Huijser,[§] Alfred R.Holzwarth,[∥] Francesco Buda,[¶] G. Agur Sevink,[¶] Huub J. M. de Groot,[¶] and Thomas L. C. Jansen[*,†]

†*University of Groningen, Zernike Institute for Advanced Materials, 9747 AG Groningen, The Netherlands.*

‡*Department of Chemistry and Hylleraas Centre for Quantum Molecular Sciences, University of Oslo, Sem Sælands vei 26, 0315 Oslo, Norway*

¶*Leiden Institute of Chemistry, Leiden University, Einsteinweg 55, 2300 RA Leiden, the Netherlands*

§*MESA+ Institute for Nanotechnology, University of Twente, Drienerlolaan 5, 7522 NB Enschede, the Netherlands*

∥*Department of Biophysical Chemistry, Max Planck Institute for Chemical Energy Conversion, Stiftstraße 34-36, 45470 Mülheim, Germany*

E-mail: t.l.c.jansen@rug.nl

CHELPG charges

Information on the partial (CHELPG) charges of atoms of the *Bchl c* molecule in the ground and the excited states, determined with the procedure described in section Quantum Chemical Parametrization are given in the separate document under the name 'CHELPG_charges.txt'

Structural analysis

Dependence of the character of excitonic states on hydrogen bonding patterns is a consequence of the difference in the local configurations of the monomers, which is evident from the structural analysis based on the histograms given in Figure S1. These 2D maps represent the correlation of the distances between centers of chromophores, given as position of Mg atoms, with the angles Θbetween the unit vectors connecting the N_A and N_C atoms in the porphyrin rings, that are parallel to the transition dipole moment of the Q_y transition.

We see 3 main peaks in the 2D maps. The first corresponds to the Mg-Mg separation of $r \approx 7.5$ Å which is the distance between the chromophores in the *syn-anti* pair. The second peak observed at $r \approx 8.3$ Å agrees with the distance between the rings of dimer units and corresponds to the distance between *syn-syn* and *anti-anti* pairs.[1] The last peak at the Mg-Mg distance of $r \approx 9.7$ Å stems from the separation between the *syn* and *anti* molecules in different rings and follows the direction of hydrogen bonding.[1]

Comparing the 2D histograms that represent structural configurations of the donor and non-donor molecules we note the main difference in the orientation of these molecules with the respect to the nearest neighbour, namely within the *syn* and *anti* pairs. The longer range packing is essentially not affected by the hydrogen bonding patterns. The representation given here is in line with previous reports on the hydrogen bonding based on molecular dynamics simulations.[2] The minimal angle Θ between the chromophores represents the case of maximal overlap of the porphyrin rings of *BChl c* molecules. This situation corresponds to the most stable ground state configuration. The structural analysis reveals that donor

molecules predominantly adopt a configuration with the smaller $\Theta \approx 10 - 20°$ angle with the neighbouring chromophores opposite to the non-donor molecules which favour the less stable configuration with $\Theta \approx 30 - 40°$.

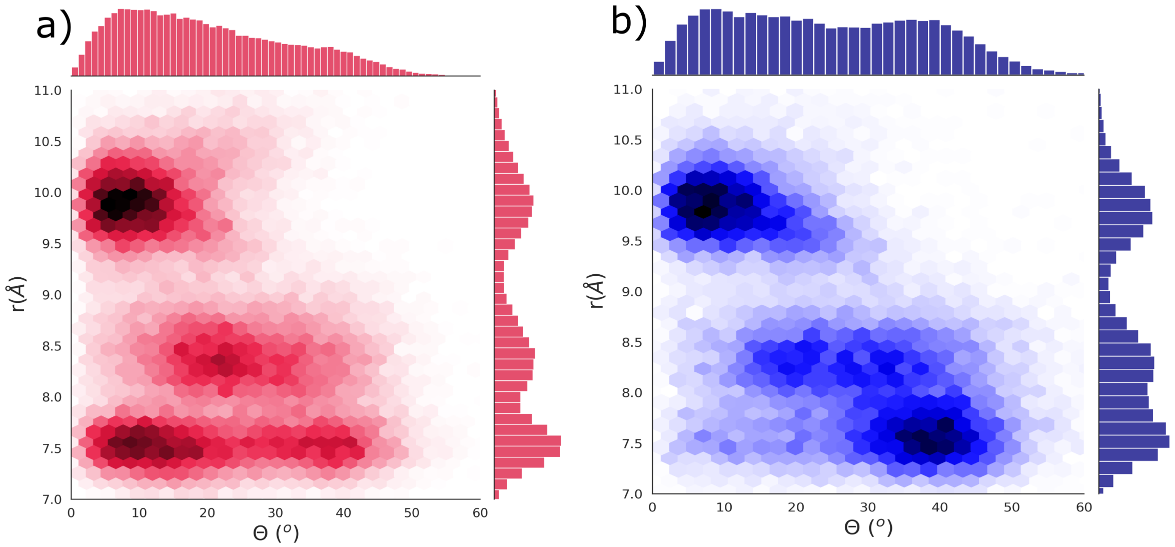

Figure S1: Structural analysis of local configurations of donor and non-donor molecules: a) 2D correlation map of the distances (r) and the angle Θ between transition dipole moment of each donor with respect to other molecules in it's neighbourhood. The right panel (b)) shows the same, but for each non-donor molecule.

2D Electronic Spectroscopy

Here, we show comparison of the linear absorption spectra of the smaller model system, used for the simulations of the 2D spectra, to the full system which consists of the three concentric cylinders (Figure S2). We also provide the 2D spectra in the initial waiting time which corresponds to the system where only molecular scale disorder is added and there is no added additional broadening coming from the higher degree of disorder (Figure S3). The additional noise/structure seen here is attributed to finite size effects and the limitation of sampling.[3]

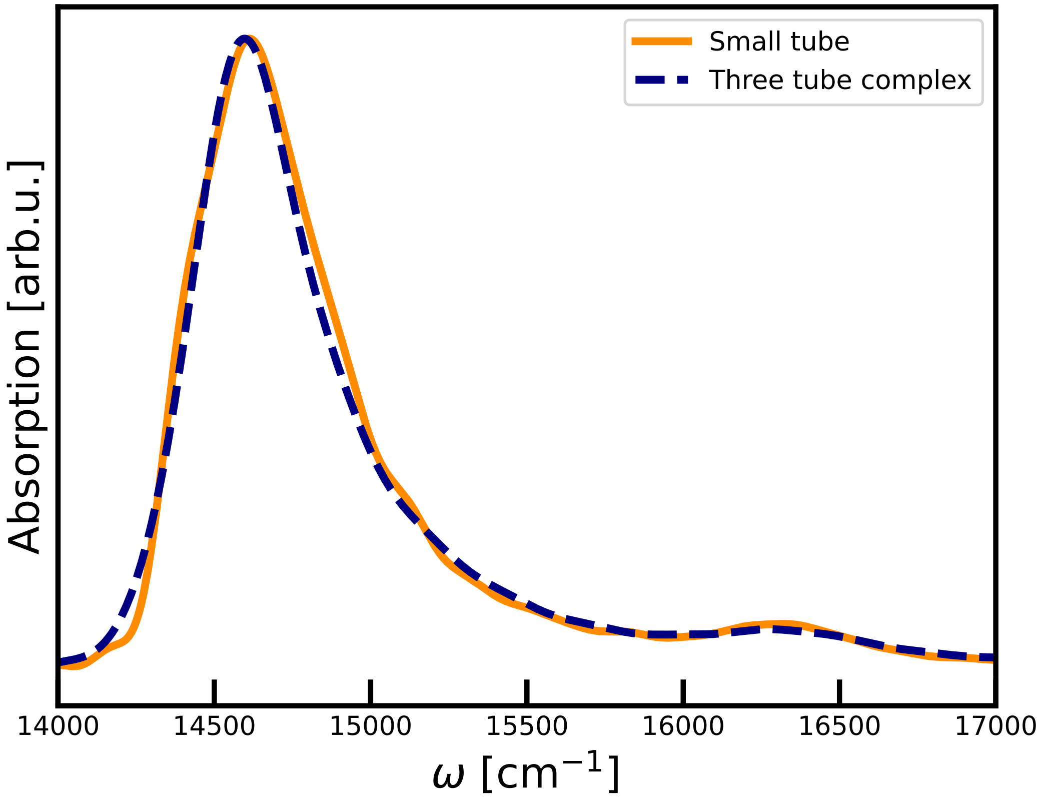

Figure S2: Comparison of the linear absorption spectrum of the three tube system compared to the model system used for simulations of the 2D spectra. Spectrum of the single tube is scaled to match relative intensity of the full system.

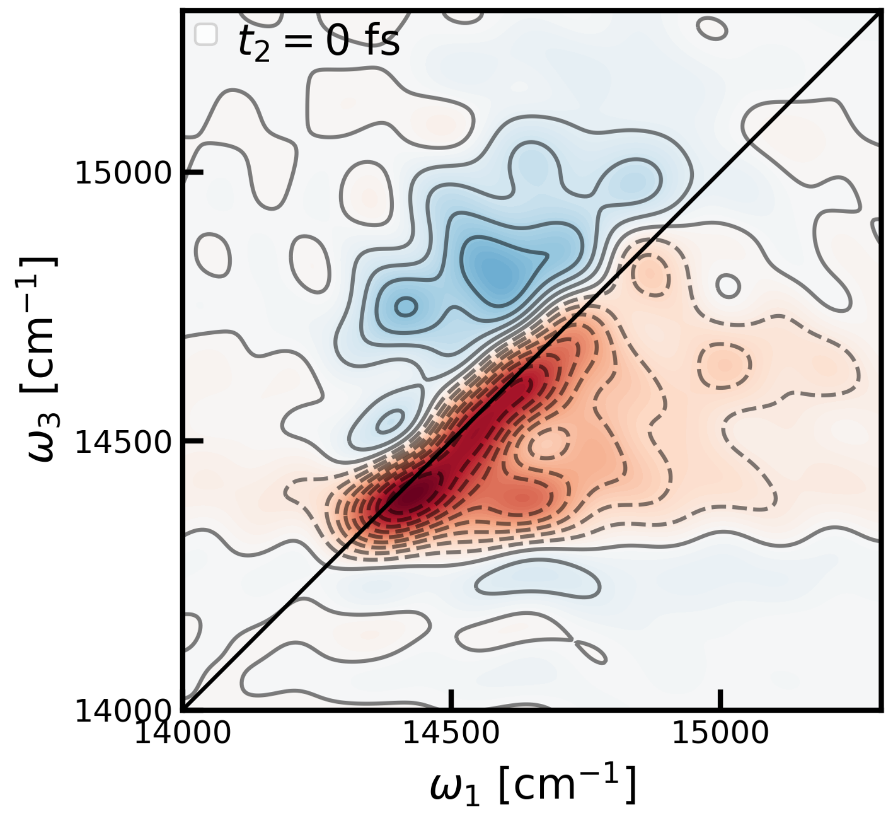

Figure S3: Simulated 2D spectrum of chlorosomes at t_2=0 waiting time without mesoscale disorder.

References

(1) Ganapathy, S.; Oostergetel, G. T.; Wawrzyniak, P. K.; Reus, M.; Gomez Maqueo Chew, A.; Buda, F.; Boekema, E. J.; Bryant, D. A.; Holzwarth, A. R.; de Groot, H. J. M. Alternating Syn-Anti Bacteriochlorophylls Form Concentric Helical Nanotubes in Chlorosomes. *Proc. Nat. Acad. Sci.* **2009**, *106*, 8525–8530.

(2) Li, X.; Buda, F.; de Groot, H. J.; Sevink, G. A. Contrasting modes of self-assembly and hydrogen-bonding heterogeneity in chlorosomes of Chlorobaculum tepidum. *J. Phys. Chem. C.* **2018**, *122*, 14877–14888.

(3) Kunsel, T.; Günther, L. M.; Köhler, J.; Jansen, T. L. C.; Knoester, J. Probing Size Variations of Molecular Aggregates inside Chlorosomes Using Single-Object Spectroscopy. *J. Chem. Phys.* **2021**, *155*, 124310.
